# Herpes zoster incidence in adults aged ≥ 20 years in Finland, 2015 to 2023: a population-based register study

**DOI:** 10.2807/1560-7917.ES.2025.30.35.2500077

**Published:** 2025-09-04

**Authors:** Aapo Juutinen, Heini Salo, Toni Lehtonen, Tuija Leino

**Affiliations:** 1 Welfare Epidemiology and Monitoring Unit, Department of Public Health, Finnish Institute for Health and Welfare, Helsinki, Finland; 2 Research Unit of Population Health, Faculty of Medicine, University of Oulu, Oulu, Finland; 3 Data Production and Methods Unit, Department of Data and Analytics, Finnish Institute for Health and Welfare, Helsinki, Finland

**Keywords:** Herpes zoster, Incidence, Finland, Shingles

## Abstract

**BACKGROUND:**

Herpes zoster, also known as shingles, is a painful skin condition caused by varicella zoster virus. Information is lacking on incidence of herpes zoster in Finland.

**AIM:**

To estimate age-specific annual incidence of herpes zoster over 9 years with data from several nationwide health registers.

**METHODS:**

In a nationwide study, we compiled a dataset encompassing the entire Finnish population by linking data from multiple population-based registers for 2015–23. The dataset includes records from nearly all healthcare providers in Finland. Case definitions were based on ICD-10 codes B02 and G53.0, and ICPC-2 code S70. The dataset was supplemented with information on the purchase of prescribed antiviral medication.

**RESULTS:**

In total, 220,693 herpes zoster cases were identified during 2015–23 among adults aged ≥ 20 years. In 2023, when register coverage was best, age-specific annual incidence rates for the entire population varied from 3.8 to 12.2, increasing with age. Incidence was higher among females than males, and highest among elderly aged ≥ 85 years living at home without organised care. Cumulative incidence data from 2023 revealed that lifetime risk of developing herpes zoster by age 85 was 42%, and as high as 46% using the incidence among elderly living at home without organised care.

**CONCLUSION:**

Herpes zoster incidence varied by sex and age group, and appeared to be under-reported in institutional and organised care settings. Currently, there is no official decision on herpes zoster vaccination in Finland, despite favourable recommendations from the national expert group and the National Advisory Committee on Vaccination.

**Key public health message**

**What did you want to address in this study and why?**

Herpes zoster, commonly called shingles, is a painful skin condition caused by the varicella zoster virus, which also causes chickenpox. In Finland, no information is available on how common herpes zoster is among different age groups. By linking data from several national health registers, we were able to estimate herpes zoster incidence among different age groups in the population aged ≥ 20 years.

**What have we learnt from this study?**

We found more than 220,000 herpes zoster cases for the period 2015–23. The risk of herpes zoster varied widely across age groups, with older people being most affected. The highest rates were estimated among those aged 85 years and older who live at home without organised care, suggesting underreporting in organised and institutional care. We estimated that by age 85, around 4 in 10 people in Finland had experienced shingles at least once in their lifetime.

**What are the implications of your findings for public health?**

Using various register information, we obtained more reliable estimates of the incidence of herpes zoster, especially among the older age groups who typically experience the most severe forms of the disease. These results support cost effectiveness studies and discussions about including shingles vaccination for adults in the national programme.

## Introduction

Herpes zoster, commonly known as shingles, is a painful skin condition caused by the varicella zoster virus (VZV). Varicella zoster virus commonly infects individuals during childhood, causing chickenpox. Following this initial infection, the virus remains latent in nerve cells. Later in life, VZV can reactivate, resulting in herpes zoster. Herpes zoster typically presents as a rash on one side of the body on one to three dermatomes, accompanied by pain and a burning sensation in the affected area. The risk of developing herpes zoster increases with age, and it has been reported that one in three people will develop the disease at some point in their lifetime [1,2]. In some cases, herpes zoster can lead to serious complications, the most common being postherpetic neuralgia, a condition characterised by long-lasting neuropathic pain. In Finland, nearly all individuals over 65 years are seropositive to VZV, i.e. at a risk of developing herpes zoster [3]. However, there is a lack of information on the incidence of herpes zoster in Finland.

Herpes zoster in elderly people (≥ 65 years) is commonly treated with antivirals, although its effect both in reducing the duration and pain of herpes zoster and preventing post-herpetic pain and other sequelae is not well shown [4,5]. However, an effective vaccine for herpes zoster exists. The first vaccine (Zostavax, Merck) came to the European market in 2006, but has since been withdrawn because of the availability of a more effective vaccine (Shingrix, GlaxoSmithKline), which was licensed in 2018 in Europe, and is the only product available on the European market. Currently, the vaccine for herpes zoster is not included in the National Vaccination Programme in Finland and vaccination is offered at one's own expense. By December 2024, there were in total around 23,000 people (0.4% of the population) in Finland who had received one of the herpes zoster vaccines, based on the Finnish National Vaccination Register.

In published review articles on herpes zoster incidence, the studies presented rely on either active or passive surveillance, mostly have a retrospective design, and generally use electronic health records from clinical registries or insurance providers as sources of cases [6-8]. The studies utilise diagnostic codes or equivalent clinical diagnoses based on symptoms, antiviral drug prescriptions, or self-reporting to identify herpes zoster cases. In larger European countries such as Germany and Netherlands, the annual incidence of herpes zoster in the whole population has been reported to vary from 3.3 to 6.0 per 1,000 persons [6,9]. When health registers documenting hospitalisations or outpatient visits to a physician are used, cases in institutions, e.g. elderly care homes, or among people living in their own home with medical or organised home care, might be under-reported. Another difficulty in incidence calculations is the need of a denominator, which might be lacking in some cases. In some studies, healthcare visits within the public sector are covered by the register used, but healthcare visits occurring in private sector are missing [10]. 

In this nationwide population-based study, we aimed to estimate the age-specific annual incidence of herpes zoster in adults in Finland by using individually linkable national registers. As the incidence of herpes zoster is usually highest among elderly people (≥ 65 years), we also used data on the institutional care and organised home care settings.

## Methods

### Study setting and population

This is an ecological study using nationwide population-based register data. In 2023, the total population of Finland was 5.6 million, 50.5% were females and 49.5% males. We obtained the age- and sex-specific population sizes from the Finnish Population Information System, an electronic national database that records individual identifiable information for all Finnish residents, such as name, sex, personal identity code, date of birth and death. In Finland, the personal identity code is used for patient identification in all healthcare registers. The Finnish healthcare system consists of a publicly funded healthcare service and a private sector accounting for one-fifth of the health expenditure in Finland [11]. The study population included all Finnish citizens aged ≥ 20 years.

### Data sources

We established a nationwide dataset including the whole Finnish population by linking data during years 2015–23 from multiple population-based registers. The data on hospitalisations and outpatient care with diagnoses referring to herpes zoster were retrieved from the Register of Primary Health Care Visits and Care Register for Health Care, both maintained by the Finnish Institute for Health and Welfare. The Register of Primary Health Care Visits contains all public primary healthcare contacts (on-site office visits, real-time and non-real-time remote contacts and phone calls) in Finland. Since 2020, it has gradually included a growing number of the private providers and occupational health service providers as well. The data from occupational health services are rather comprehensive by 2023, but the data from private providers remain less comprehensive. The Care Register for Health Care (referred to also as secondary healthcare) contains data on inpatient care, and specialised outpatient care. Since 2020, the private providers began contributing data in the register. Data from the Register of Primary Health Care Visits and the Care Register for Health Care included date of entry, healthcare sector, main and secondary diagnoses and form of care, e.g. inpatient care, outpatient care, home care, etc.

Prescriptions and purchases of antiviral therapy were retrieved from the Prescription Centre database maintained by the Social Insurance Institution of Finland. The database includes all electronic prescriptions and pharmacy dispensing details from 2017 onwards. Data from the Prescription Centre database included date of prescription and purchase, ATC-code, strength of the drug, package size and purpose of use. 

We used data from the Care Register of Social Welfare (2015–23) regarding social care services, i.e. institutional care and assisted living, to identify individuals in institutional care and housing services. Data from the Care Register of Social Welfare included date of entry and exit and form of care. Individuals in home care were identified from the Register of Primary Health Care Visits based on the data about the use of home care services.

### Case definition

Herpes zoster case was defined as a person (i) using healthcare services with International Classification of Diseases, Tenth Revision (ICD-10) codes B02 (Zoster) and G53.0 (Postherpetic neuralgia) or International Classification of Primary Care, 2nd edition (ICPC-2) code S70 (Herpes zoster) as the primary or secondary diagnosis or (ii) purchasing defined prescribed antiviral medication where the strength and package size of the medication indicated a dosage used for the treatment of herpes zoster. Detailed information about the included medication is given in Supplementary Table S1. We defined the cases by the ‘purpose of use' variable, which is an open text field. We included all prescriptions containing words referring to herpes zoster (n = 37,973) or with missing information (n = 55,238). Prescriptions with missing information about the purpose of use were included in the analysis because we considered these to be most likely intended for the treatment of herpes zoster based on the included strength and package size of the medication. We excluded prescriptions when the variable contained text that did not refer to shingles (n = 25,047). The time point of the index event was determined by the individual's earliest recorded herpes zoster diagnosis or antiviral medication purchase, whichever occurred first. To ensure that only each individual’s first herpes zoster case (incident case) was captured, we applied a washout period from 2012 to 2014 to exclude individuals that had any herpes zoster related events before 2015.

### Statistical analysis

We assessed the annual incidence (per 1,000 people) of herpes zoster and herpes zoster-related inpatient care from 2015 to 2023 in adults aged ≥ 20 years. The annual incidence rates stratified by age and sex were calculated by dividing the number of cases by population size estimates. The cases identified by a purchase of antiviral medication with herpes zoster treatment dosage independently of a recorded healthcare service use with a herpes zoster diagnosis were reported separately.

The cases were categorised according to the healthcare sector in which the index event was identified. The healthcare sector involved was not known for cases identified through the purchase of prescribed antiviral medication. In elderly people aged ≥ 65 years, the number of cases and annual incidence rates in 2017–23 were further assessed separately for three groups: (i) those living at home without organised social and healthcare services and (ii) those living at home with organised home care services and (iii) those in institutional or residential care. An organised home care client was defined as a person who had at least one home care visit in the 61 days before the herpes zoster diagnosis. Likewise, a person was considered an institutional or residential care client if herpes zoster was diagnosed during a stay at an institutional care facility.

All the statistical analysis and figures were performed using RStudio (R version 4.4.1). The figures were created using the R package ggplot2.

## Results

### Herpes zoster cases and incidence

A total of 220,693 herpes zoster cases were identified in Finland in 2015–23 among adults aged ≥ 20 years (Table 1). Of these, 100,434 cases were identified from the Prescription Centre database, 90% (n = 90,281) of which were not found in other registers and would therefore have remained completely unidentified. A more detailed overview of the cases is presented in Supplementary Table S2 and Supplementary Table S3.

**Table 1:** Numbers of herpes zoster cases from respective registries, Finland, 2015–2023 (n = 220,693)

Year	Numbers of herpes zoster cases by registry			Total cases
	Register of primary health care visits^a^	Care register for health care^b^	Prescription centre database^c^	
2015	7,762	2,018	11,908	21,688
2016	7,724	2,239	12,148	22,111
2017	7,849	2,445	13,896	24,190
2018	7,974	2,571	13,403	23,948
2019	7,803	2,885	13,779	24,467
2020	10,352	2,681	11,529	24,562
2021	14,148	3,461	8,053	25,662
2022	14,792	3,241	7,879	25,912
2023	17,327	2,987	7,839	28,153
Total	95,731	24,528	100,434	220,693

^a^ Public primary healthcare, occupational healthcare and private primary healthcare.

^b^ Secondary healthcare.

^c^ Purchase of prescribed antiviral medication.

Numbers of herpes zoster cases (≥ 20 years) categorised according to the registry from which they were identified. Cases identified from the Register of Primary Health Care Visits start to increase from 2020, when private providers and occupational health service providers began contributing data.

During the study period, the cases identified from the Prescription Centre database decreased and the cases identified from the Register of Primary Health Care Visits increased as the private providers and occupational health service providers began contributing data to the latter register in 2020. In 2015–19, 56% of cases were identified from the Prescription Centre database and 34% from the Register of Primary Health Care Visits. In 2020–23, the proportions were reversed (34% and 54%, respectively). The proportion of cases identified in secondary healthcare remained stable throughout the study period (9–13%).

**Figure 1:**
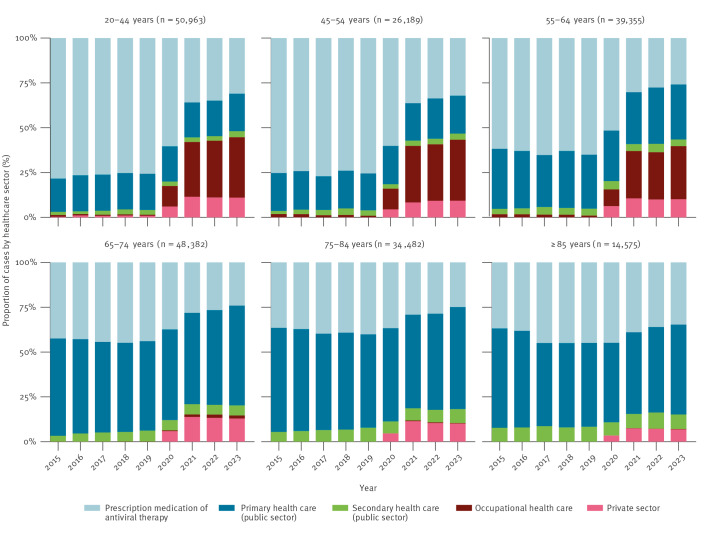
Distribution across healthcare sectors for identified herpes zoster cases by year and age group, Finland, 2015–2023 (n = 213,946)

Furthermore, more comprehensive primary healthcare register data changed the distribution of healthcare sectors in which cases were identified during the study period. In 2023, one third of the cases among the working-aged adults (20–64 years) were identified in occupational healthcare (Figure 1). Among elderly people aged ≥ 65 years, cases were predominantly identified in public primary healthcare, with proportions ranging from 50% to 56% in 2023. The proportion of cases identified in private healthcare remained low during the entire study period as the data improvements were more limited. The proportion of secondary healthcare cases remained low because the registry data have been comprehensive during the whole study period.

For cases identified through the purchase of prescribed antiviral medication, the healthcare sector involved was not known. The distributions excluded 6,747 (3%) cases with recorded healthcare service use with a herpes zoster diagnosis, but without information of the healthcare sector. The n numbers indicate the total number of herpes zoster cases in each age group.

**Figure 2:**
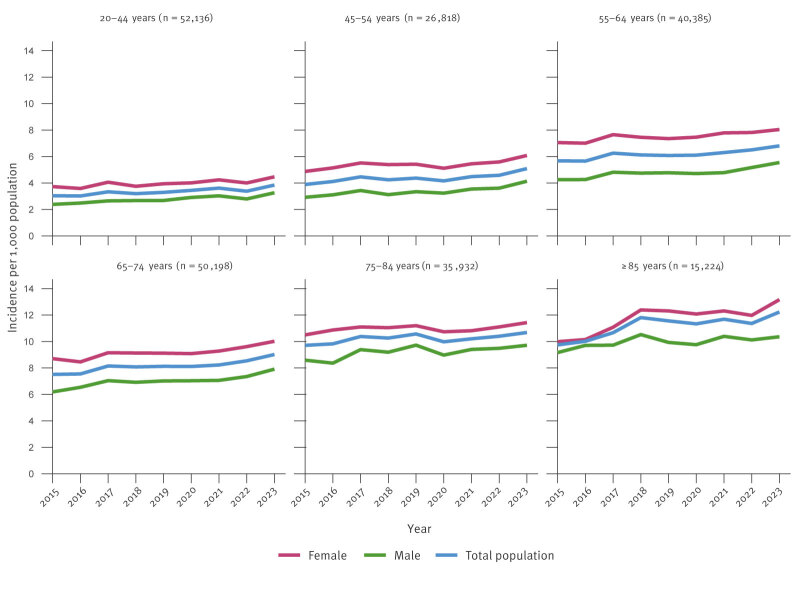
Annual incidence of herpes zoster per 1,000 population by sex and age group, Finland, 2015–2023 (n = 220,693)

The annual incidence of herpes zoster increased during the study period because of the improved coverage of registers, and because of the ageing population. The annual incidence increased by age and was higher in females than in males across all age groups (Figure 2). Among females, the annual incidence rates (per 1,000 population) in 2023 were 4.5, 6.1, 8.0, 10.0, 11.4 and 13.2 for the age groups 20–44, 45–54, 55–64, 65–74, 75–84 and ≥ 85 years, respectively, compared with 3.3, 4.1, 5.5, 7.9, 9.7 and 10.4 for males in the same age groups. For the entire population in 2023, the annual incidence rates were 3.8, 5.1, 6.8, 9.0, 10.7, and 12.2 for the age groups 20–44, 45–54, 55–64, 65–74, 75–84, and ≥ 85, respectively. The incidences within total population are presented in more detail in Supplementary Table S2.

Incidences are shown for females (red line), males (green line) and the total population (blue line). The n numbers indicate the total number of herpes zoster cases in each age group. The incidences in the total population are presented in more detail in Supplementary Table S2.

**Figure 3:**
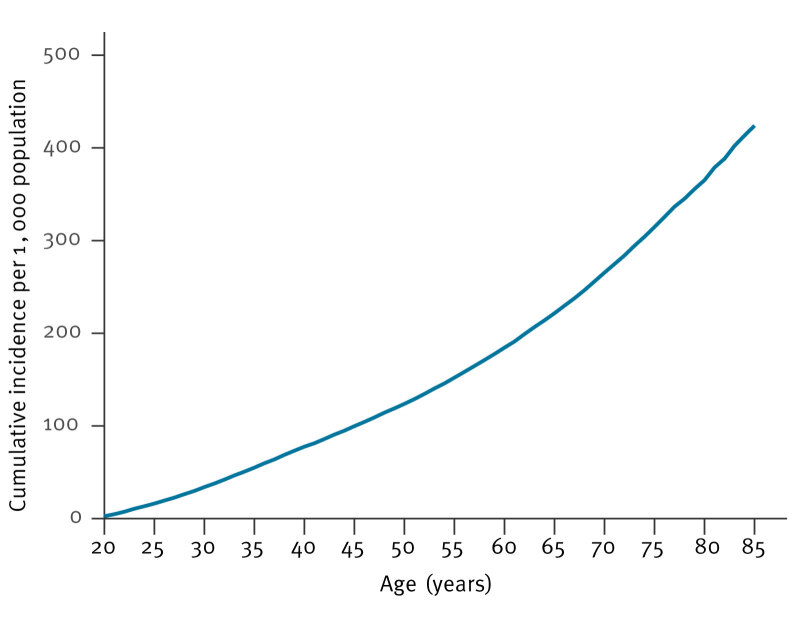
Cumulative incidence of herpes zoster per 1,000 population, Finland, 2023 (n = 26,451)

Cumulative incidence data from 2023 revealed that the lifetime risk of developing herpes zoster by the age of 85 years was 42% (Figure 3). Notably, the risk began to rise markedly after age 60 years, and around 56% of the cases occurred among people aged ≥ 60 years (see Supplementary Table S2).

### Herpes zoster incidence in inpatient care

The proportion of cases needing inpatient care for herpes zoster was calculated by age, irrespective of the mode of identifying the herpes zoster cases, i.e. where the primary contact has been recorded. The proportion of hospitalised cases and the corresponding annual incidence rates were higher in older age groups. Among herpes zoster cases aged under 65 years, ca 1% required inpatient care in 2023, corresponding to annual incidence rate of 0.05 per 1,000 population (Table 2). The proportion of cases requiring inpatient care increased to 2.1% among those aged 65–74 years, to 3.3% among those aged 75–84 years and to 7.2% among those aged ≥ 85 years. The corresponding annual incidence rates were 0.2, 0.4 and 0.9 per 1,000 population.

**Table 2:** Proportion of hospitalised herpes zoster cases by age and corresponding annual incidence rates per 1,000 people, Finland, 2015–2023 (n = 6,967)

Year	Age group											
	20–44 years		45–54 years		55–64 years		65–74 years		75–84 years		≥ 85 years	
	%	Incidence	%	Incidence	%	Incidence	%	Incidence	%	Incidence	%	Incidence
2015	1.4	0.04	2.0	0.1	2.4	0.1	3.2	0.2	7.0	0.7	14.7	1.4
2016	0.9	0.03	2.1	0.1	2.5	0.1	3.5	0.3	6.9	0.7	13.8	1.4
2017	1.2	0.04	1.4	0.1	2.3	0.1	2.9	0.2	6.5	0.7	13.0	1.4
2018	1.3	0.04	1.5	0.1	2.3	0.1	3.4	0.3	6.3	0.6	12.7	1.5
2019	1.3	0.04	1.8	0.1	2.3	0.1	3.5	0.3	6.7	0.7	11.6	1.3
2020	1.2	0.04	1.5	0.1	1.9	0.1	2.8	0.2	5.3	0.5	9.8	1.1
2021	0.9	0.03	1.1	0.05	1.9	0.1	2.9	0.2	5.3	0.5	11.7	1.4
2022	0.9	0.03	1.1	0.05	1.2	0.1	2.3	0.2	4.2	0.4	8.8	1.0
2023	1.1	0.04	1.0	0.05	1.1	0.1	2.1	0.2	3.3	0.4	7.2	0.9
Total	1.1	0.04	1.5	0.1	2.0	0.1	2.9	0.2	5.6	0.6	11.3	1.3

### Herpes zoster cases and incidence among elderly people

**Figure 4:**
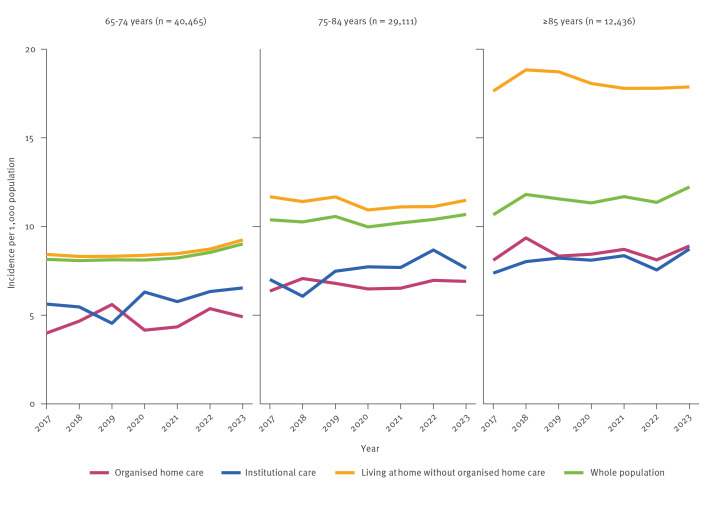
Annual herpes zoster incidence in elderly people by calendar year and age group, Finland, 2017–2023 (n = 82,012)

To understand whether cases occurring in social care settings are reported in national registers as well as in healthcare registries, we assessed annual incidence rates in both institutional and organised home care separately in three age groups (65–74, 75–84, ≥ 85 years). In the age groups of 65–74 and 75–84 years, annual incidence rates were higher in institutional care, with rates of 6.5 and 7.7 (per 1,000 population) in 2023, compared with 4.9 and 6.9 in organised home care for the corresponding age groups (Figure 4). However, in the oldest age group (≥ 85), the annual incidence was higher among those in organised home care, with a rate of 8.9 compared with 8.7 in institutional care. The annual incidence in the whole population was higher than in either institutional or organised home care settings. The highest annual incidence rates were observed among those living at home independently without organised home care, with rates of 9.2, 11.5, and 17.9 in 2023 for the age groups 65–74, 75–84, and ≥ 85, respectively.

Incidences per 1,000 population are split by those without organised social and healthcare services (yellow line), those living at home with organised home care services (red line), those in institutional or residential care (blue line), whole population (green line) and by calendar year and age group. The 2015–16 data were excluded because of lack of data. The n number indicates the total number of herpes zoster cases in the age group. The incidences in more detail are presented in Supplementary Table S4.

## Discussion

Our study used nationwide, population-based health registers to identify herpes zoster cases. These registers cover the entire population of Finland, enabling us to assess the most accurate estimates for herpes zoster annual incidence rates for people ≥ 20 years. The quality and reliability of these register data have been found to be good [12]. Additionally, the registers include nearly all healthcare services in Finland, covering comprehensively the public and occupational health sectors, and partially the private sector. 

The increasing incidence of herpes zoster with age is associated with a declining immune system [13]. Our data showed that the incidence was highest among elderly people ≥ 85 years living at home without organised care. It is, therefore, unlikely that older adults living at home without organised home care—who are generally healthier and more functional—would have a higher prevalence of herpes zoster than more frail, elderly individuals residing in institutions. The explanation is more likely that cases identified in long-term institutional care, for example, are treated alongside other illnesses and remain unregistered in official records. Our results thus support the fact that herpes zoster appears to be under-reported in institutional and home care settings. If the incidence detected among elderly people not needing organised care is assumed to be the real age-specific incidence, the lifetime risk for developing herpes zoster would be as high as 46% by the age of 85. Therefore, it is crucial to be able to estimate the age-specific incidence as accurately as possible, especially among elderly people ≥ 65 years, to better tailor prevention strategies for this vulnerable population.

Analysis of age-specific annual incidence by sex revealed that herpes zoster incidence is higher among females than males, particularly in the age group of 55–64 years. A higher risk of developing shingles among women has been observed previously [14-16], although the reason for this phenomenon is not entirely clear. To some extent, this could be explained by women using healthcare services more frequently, and since incidence rates in various studies are based on healthcare records, the cases among women could be documented more comprehensively than those among men [17,18]. Other explanations could be increased frequency of immunosuppressive diseases or medications, and genetic or hormonal factors. Based on full-text medical records allowing proper adjusting, we conclude sex to be an independent risk factor [16].

In general, estimated age-specific incidences in this study are roughly two times higher compared to the previous study that used only register data on public primary healthcare visits in Finland [10]. This is most likely due to the fact that we used more comprehensive register data with the addition of data on prescribed antiviral therapies. For example, data on occupational health and the private sector were unavailable at the time of the earlier studies.

In 2013, before herpes zoster vaccinations were included in the United Kingdom (UK) national vaccination programme, the incidence estimates based on primary care general practitioners were highest for those over 80 years old [19], being roughly 10 per 1,000, i.e. lower than estimated in our study. In the United States, data from insurance databases showed pre-vaccination era (before 2006) incidences very similar to those in the UK [20]. Furthermore, in two separate Swedish studies published in 2013 and 2015, in which the incidence was estimated based on purchased antiviral medications, estimated incidences were slightly lower than in our study [7,21]. A study from 2020 using primary and secondary healthcare data in Norway showed similar to lower estimates, especially among the very old [8]. Combining multiple registers, as in our study and in a German study from 2015, yielded higher incidence rates: 14 per 1,000 in Germany and, up to 12.2 in ours [6].

In population surveys, approximately one-third of older adults have reported having had shingles at least once [1,2]. Based on data from Canadian and British outpatient visits, it has been estimated that 28% and 30% of people, respectively, have developed shingles by the age of 80 years [22]. When more comprehensive register data are utilised, as in our study, the cumulative incidence was found to be ca 37% by the age of 80 and over 40% by the age of 85 years. Furthermore, if we assume that the risk of getting herpes zoster is similar irrespective of where the people are living, i.e. including incidence of elderly people living at home without organised care, the cumulative incidence is even higher, at 46%. As we have used nationwide data on the healthcare visits coded as herpes zoster and data on purchase of prescribed antiviral medications, we assumed that we have reached cases with any healthcare contacts. However, the share of cases without any contact to healthcare should be added to our estimate of cumulative incidence during lifetime.

By December 2024, there were in total 23,000 people in Finland who had received one of the herpes zoster vaccines since the year 2006, the year when the vaccine first came to the European market. This vaccination effort may have contributed to a decrease in the incidence of the herpes zoster. However, considering that the number of vaccinated individuals is limited and distributed over time, and that the effectiveness and the duration of protection obtained by the earlier used Zostavax vaccine was limited [23,24], the overall impact on incidence is likely to be minor. In Finland, no official decision has yet been made on herpes zoster vaccination, even though it has been recommended by both the national expert group and the National Advisory Committee on Vaccination.

There are a few limitations in our study. Firstly, since data from the occupational health and private sectors were only available in the registers starting in 2020, we were unable to calculate herpes zoster cases or incidence rates related to these sectors for earlier years. Thus, because of the gradual improvement in data completeness over time, we were unable to derive a reliable estimate of the trend in herpes zoster incidence. Secondly, some records contained missing or incorrectly recorded information regarding the healthcare sector (around 3% of the cases), making it impossible to classify all cases by sector. Thirdly, data on institutional care were partially incomplete, as the register is updated annually, and the 2023 data were not fully available at the time of the study. Also, since the data on home care were lacking from the years 2015–16, we excluded these years from the results. Fourthly, this was the first time we assessed herpes zoster incidence using data on antiviral prescriptions. The data contain information in free text format, which is also partly incomplete. Thus, it is difficult to do fully comprehensive data extractions. Additionally, drug prescriptions do not always correspond directly to coded diagnoses, as medications are often used in preventive settings as well. If antiviral medications are used in large quantities for prevention of herpes zoster, or for treatment of varicella or herpes simplex virus, our estimates would be too high. However, we have included only prescriptions with high dosages, not generally used for preventive purposes or for treatment of herpes simplex, except for severe and rare forms such as herpes simplex virus meningitis. Also, we only included prescriptions where the purpose of use in open text field referred specifically to the herpes zoster, i.e. contained any word referring to herpes zoster, or the information was completely missing. Nilsson et al. used the same medication dosages with slightly different exclusion criteria, regarding the wording in the purpose of use of the medication [7]. As a result, 12% of the antiviral prescriptions were excluded from the analysis. Assuming correspondingly that ca 12% of our prescription data with completely missing information about the purpose of use include incorrect herpes zoster cases, this would result in 3% of overestimated cases in our data.

## Conclusions

By linking data at individual level from different health registers, we were able to reach a more reliable estimate of the herpes zoster incidence in Finland. Our study showed that the incidence of herpes zoster is varying by sex and age group, being higher in women than in men and increasing with age. Herpes zoster appears to be under-reported in institutional and home care settings. As a follow-up to this study, our aim is to estimate age-specific cost variables to study the cost-effectiveness of different vaccination strategies. Also, future research should focus on examining the complications and risk factors associated with the disease in more detail.

## Data Availability

By Finnish law, the authors are not permitted to share individual-level register data (Section 24, 'Confidential Official Documents’, of the Finnish Act on the Openness of Government Activities (621/1999), available from: https://www.finlex.fi/api/media/statute-foreign-language-translation/688690/mainPdf/main.pdf?timestamp=1999-05-20T21%3A00%3A00.000Z). Aggregated data can be found in the Supplementary materials.

## Supplementary Data

197000 Supplement
