# Supplementary material for: Herpes zoster incidence in adults aged ≥ 20 years in Finland, 2015 to 2023: a population-based register study
Source: Euro Surveill. 2025 Sep 4;30(35):2500077. doi: 10.2807/1560-7917.ES.2025.30.35.2500077 (PMC12413605; doi:10.2807/1560-7917.ES.2025.30.35.2500077)
Supplement: Supplement [file 25-00077_JUUTINEN_SUPPLEMENT.pdf]

Supplementary material

This supplementary material is hosted by *Eurosurveillance* as supporting information alongside the article *Herpes zoster incidence in adults aged ≥ 20 years in Finland, 2015 to 2023: a population-based register study*, on behalf of the authors, who remain responsible for the accuracy and appropriateness of the content. The same standards for ethics, copyright, attributions and permissions as for the article apply. Supplements are not edited by *Eurosurveillance* and the journal is not responsible for the maintenance of any links or email addresses provided therein.

Table S1. Antiviral prescription medication used in the treatment of herpes zoster.

ATC-code (active substance)	Strenght of the drug	Package size
J05AB01 (Aciclovir)	800 mg	35 fol
J05AB11 (Valaciclovir)	500 mg	42 fol

Table S2. Number of cases and incidence (/1000) of herpes zoster in Finland.

Number of cases									
**Age group**	**2015**	**2016**	**2017**	**2018**	**2019**	**2020**	**2021**	**2022**	**2023**
20-24	789	712	851	784	727	800	772	700	819
25-29	1003	1014	1104	1112	1126	1141	1220	1072	1136
30-34	1127	1132	1196	1170	1199	1367	1358	1389	1575
35-39	1125	1177	1313	1200	1353	1333	1458	1336	1670
40-44	1128	1130	1262	1244	1282	1309	1447	1388	1586
45-49	1295	1280	1353	1222	1207	1216	1310	1339	1670
50-54	1524	1652	1761	1664	1721	1551	1660	1689	1704
55-59	1895	1862	2111	2036	2066	2025	2112	2152	2286
60-64	2304	2308	2468	2437	2363	2400	2431	2523	2606
65-69	2821	2670	2871	2822	2740	2692	2685	2804	2910
70-74	2005	2237	2650	2776	3000	3052	3098	3119	3246
75-79	1868	2033	2140	2059	2186	2154	2403	2624	3038
80-84	1447	1473	1551	1679	1762	1766	1866	1969	1914
≥85	1357	1431	1559	1743	1735	1756	1842	1808	1993
**Incidence**									
**Age group**	**2015**	**2016**	**2017**	**2018**	**2019**	**2020**	**2021**	**2022**	**2023**
20-24	2.3	2.1	2.6	2.4	2.3	2.6	2.5	2.3	2.7
25-29	2.9	2.9	3.1	3.1	3.2	3.2	3.5	3.1	3.3
30-34	3.2	3.2	3.4	3.3	3.4	3.8	3.8	3.7	4.2
35-39	3.3	3.4	3.7	3.4	3.8	3.7	4.0	3.7	4.5

40-44	3.5	3.5	3.8	3.6	3.7	3.8	4.1	3.9	4.3
45-49	3.7	3.8	4.1	3.9	3.8	3.8	4.0	4.0	4.8
50-54	4.1	4.4	4.8	4.6	4.8	4.5	4.9	5.2	5.4
55-59	5.1	5.1	5.8	5.5	5.6	5.5	5.8	5.9	6.3
60-64	6.2	6.2	6.7	6.7	6.6	6.7	6.9	7.1	7.3
65-69	7.4	7.1	7.8	7.8	7.7	7.6	7.6	8.0	8.4
70-74	7.7	8.1	8.6	8.4	8.6	8.6	8.9	9.1	9.6
75-79	9.5	9.6	10.3	9.8	10.3	9.3	9.8	9.6	10.3
80-84	10.0	10.1	10.4	10.8	10.9	11.0	10.8	11.7	11.3
≥85	9.7	10.0	10.7	11.8	11.6	11.3	11.7	11.4	12.2

Table S3. The cases identified by a purchase of antiviral medication with herpes zoster treatment dosage and without recorded healthcare service use with a herpes zoster diagnosis. Number of cases and incidence (/1000).

Number of cases									
**Age group**	**2015**	**2016**	**2017**	**2018**	**2019**	**2020**	**2021**	**2022**	**2023**
20-24	481	419	503	466	437	418	236	254	238
25-29	710	693	767	745	743	659	411	367	344
30-34	824	809	844	811	861	698	428	412	437
35-39	832	849	968	853	965	751	479	436	492
40-44	820	835	946	876	934	737	488	463	482
45-49	893	887	963	858	834	664	453	415	510
50-54	971	1052	1192	1058	1139	793	521	514	509
55-59	1086	1116	1322	1239	1297	982	565	553	589
60-64	1098	1144	1323	1275	1234	1026	628	596	583
65-69	996	972	1075	1120	1057	889	638	710	634
70-74	612	708	933	975	1017	902	709	696	680
75-79	489	576	668	643	702	620	550	607	632
80-84	414	395	465	540	550	544	462	506	423
≥85	378	435	558	625	640	657	601	562	613
**Incidence**									
**Age group**	**2015**	**2016**	**2017**	**2018**	**2019**	**2020**	**2021**	**2022**	**2023**
20-24	1.4	1.3	1.5	1.5	1.4	1.4	0.8	0.8	0.8
25-29	2.1	2.0	2.2	2.1	2.1	1.9	1.2	1.1	1.0
30-34	2.3	2.3	2.4	2.3	2.5	2.0	1.2	1.1	1.2
35-39	2.4	2.4	2.8	2.4	2.7	2.1	1.3	1.2	1.3
40-44	2.6	2.6	2.8	2.6	2.7	2.1	1.4	1.3	1.3
45-49	2.5	2.6	2.9	2.7	2.7	2.1	1.4	1.2	1.5
50-54	2.6	2.8	3.2	2.9	3.2	2.3	1.5	1.6	1.6
55-59	3.0	3.1	3.6	3.4	3.5	2.7	1.5	1.5	1.6
60-64	3.0	3.1	3.6	3.5	3.4	2.9	1.8	1.7	1.6
65-69	2.6	2.6	2.9	3.1	3.0	2.5	1.8	2.0	1.8

70-74	2.3	2.6	3.0	2.9	2.9	2.5	2.0	2.0	2.0
75-79	2.5	2.7	3.2	3.1	3.3	2.7	2.2	2.2	2.2
80-84	2.9	2.7	3.1	3.5	3.4	3.4	2.7	3.0	2.5
≥85	2.7	3.0	3.8	4.2	4.3	4.2	3.8	3.5	3.8

Table S4. Number of cases and incidence (/1000) of herpes zoster in different social care settings.

**Number of cases**								
**Social care setting**	**Age group**	**2017**	**2018**	**2019**	**2020**	**2021**	**2022**	**2023**
Organised home care	65-74	141	166	202	163	154	173	147
	75-84	396	433	411	420	408	426	398
	≥85	466	529	480	520	528	482	512
Institutional care	65-74	61	61	51	67	57	61	62
	75-84	198	173	209	210	207	236	215
	≥85	338	372	373	366	365	331	386
Living home without organised home care	65-74	5319	5371	5487	5514	5572	5689	5947
	75-84	3097	3132	3328	3290	3654	3931	4339
	≥85	755	842	882	870	949	995	1095
Whole population	65-74	5521	5598	5740	5744	5783	5923	6156
	75-84	3691	3738	3948	3920	4269	4593	4952
	≥85	1559	1743	1735	1756	1842	1808	1993
**Incidence**								
**Social care setting**	**Age group**	**2017**	**2018**	**2019**	**2020**	**2021**	**2022**	**2023**
Organised home care	65-74	4.0	4.7	5.6	4.2	4.3	5.4	4.9
	75-84	6.4	7.1	6.8	6.5	6.5	7.0	6.9
	≥85	8.1	9.4	8.3	8.4	8.7	8.1	8.9
Institutional care	65-74	5.6	5.5	4.5	6.3	5.8	6.3	6.5
	75-84	7.0	6.1	7.5	7.7	7.7	8.7	7.7
	≥85	7.4	8.0	8.2	8.1	8.4	7.6	8.7
Living home without organised home care	65-74	8.4	8.3	8.3	8.4	8.5	8.7	9.2
	75-84	11.7	11.4	11.7	10.9	11.1	11.1	11.5
	≥85	17.6	18.8	18.7	18.1	17.8	17.8	17.9
Whole population	65-74	8.1	8.1	8.1	8.1	8.2	8.5	9.0
	75-84	10.4	10.3	10.6	10.0	10.2	10.4	10.7
	≥85	10.7	11.8	11.6	11.3	11.7	11.4	12.2
